# Innovation for a greener future: Disentangling the asymmetric impact of green finance on green technology in european union economies

**DOI:** 10.1016/j.heliyon.2024.e30129

**Published:** 2024-04-22

**Authors:** Jian Yuan, Sajid Ali, Raima Nazar, Muhammad Imdad Ullah

**Affiliations:** a Visual Computing and Virtual Reality Key Laboratory of Sichuan Province, Sichuan Normal University, Chengdu, 610066, China; b School of Economics, Bahauddin Zakariya University, Multan, Pakistan; c Department of Economics, The Women University, Multan, Pakistan

**Keywords:** Green technology innovation, Quantile-on-Quantile estimation, Green finance

## Abstract

Green finance has originated as a critical factor for green development, where green technology innovation is a primary approach. Yet, limited information is available regarding how green finance influences green technological creation. This work probes the asymmetric linkage between green finance and green technology innovation in the selected European Union countries (Germany, Sweden, France, Italy, Netherlands, Spain, Denmark, Norway, Belgium, and Finland). Prior works adopted panel data methods to get usual outcomes concerning the green finance-green technology nexus, nevertheless the reality is that various nations did not establish such connection separately. The present work, in contrast, utilizes a peculiar tool, 'Quantile-on-Quantile,' that facilitates research to probe temporal reliance in entire nations by giving global yet economy-specific intuitions on the variables’ correlation. Estimates exhibit that green finance enhances green technology innovation in almost all our chosen nations at definite quantiles of data distribution. Furthermore, the findings expose that the extent of asymmetry throughout various quantiles of our variables differs by the economy, emphasising the relevance of policymakers paying particular consideration while employing green innovation and green finance policies.

## 1 Introduction

Green finance (GF) has gained a lot of interest during the past few decades. GF indicates investment and financing operations that create benefits related to the environment, reduce environmental hazards, and show green behaviors [1]. The core mission of GF is to give financial services regarding growth of a green economy using various cutting-edge and specialized financial products [2]. According to research, when businesses invest in green technology research and development, such as carbon emission reduction, flexible financing mechanisms, including angel investment, private equity, and green bonds (GB), can help businesses secure GF [3]. Furthermore, these financial resource providers will provide expert advice to the green venture and aid them in completing the transition to digital operation and cleaner manufacturing ([4]; [3]; [5]).

It is widely recognized that people worldwide share the aspiration to create an economy fuelled by innovation and embark on a trajectory of sustainable growth [1]. GTI is a technical innovation for resource conservation and environmental preservation [6]. For businesses, implementing green technology would benefit both long-run financial and environmental performance by lowering environmental protection costs. GTI may not only provide economic value by leveraging the effect of innovation, but it can also add environmental value by enhancing production and lowering energy usage [7,8]. It contributes to creating an effective, low-carbon, clean, and green production system, which is required for long-run green development. Meanwhile, GTI is a high-investment and high-risk activity that requires a significant amount of external funding. However, many firms carrying out GTI face issues due to current technical spillover effects, difficulty in quantifying investment value, delayed investment return period, and so on, resulting in asymmetrical information [4,9].

It is difficult to probe the GF-GTI association. Is it correct that GF increases GTI in European Union (EU) economies? Is there an asymmetric bond between GF and GTI? How does this connection transform between nations when data points are distributed differently? What are the policy repercussions of GTI variations due to changes in GF? As per available literature, these are unsolved issues, and few empirical analyses address the previously mentioned challenges. The study's contribution to earlier literature might be categorized into various classifications: Various empirical analyses on the bond between GF and GTI have been in current years (for instance, Ref. [6,4]; Yu et al., executed 2021; [10]). To the foremost of our perception, no preceding research on the GF-GTI link in EU economies has been conducted. Earlier investigations concentrated on panel data techniques to establish the GF-GTI link despite the reality no evidence of such bond was witnessed in several other nations. In the present paper, the QQ tool was utilized to give international yet economy-specific comprehension regarding the GF-GTI association. The QQ tool assesses every nation's temporal reliance on an individualized basis. Numerous features of the GF-GTI nexus create it complicated to explore utilizing typical econometric approaches (i.e., OLS and traditional quantile regression). Because standard parametrical estimates are responsive to diversions and fail to account for slope heterogeneity, they do not adequately capture the influence of GF on GTI [11,12]. Therefore, it is necessary to employ a convincing econometric methodology, in the same way as QQ, that is flexible to variations and capable of addressing varying slopes in order to evaluate the influence of GF on GTI [11]. Past research analysed definite parameters (neutral, positive, or inverse) throughout the entire data distribution sample. The present work, conversely, depicts that distinctive indications (either inverse or positive) may be attained with a great extent of quantiles. GF might have a diverse influence when the economy is in a depression as opposed to while it is growing. Similarly, higher ranks of GF might affect GTI differently than lesser levels. As the severity of GF grows, the relationship between GF and GTI might become additional intricate and vibrant. We expect the non-linear GF-GTI connection due to distributed characteristics to prompt broken-off oscillations in economic elements [12]. Given the dynamic nature of the relationship between GF and GTI, our approach focusing on individual countries can offer valuable, country-specific recommendations to policymakers and governments. These recommendations aim to assist in achieving economic, social, and political objectives, whether at low, higher, or intermediate GF-GTI levels.

The ongoing study concentrates on EU economies for a variety of grounds. First, our chosen economies are harming the environment because of their conviction on typical energy sources [13]. Second, our opted EU nations' features are linked by their common political, social, and economic characteristics. Many countries encourage economic integration because it fosters economic, social, and financial development. History has shown that financial initiatives and technological innovation in an economy may quickly expand to adjacent countries. In addition, the financial sector of a nation is dependent on the financial sector of its adjacent nations, together with internal and external shocks [13]. Third, we adopt the QQ approach, known as the time series econometric approach, which studies every economy distinctly to address cross-sectional dependence and slope heterogeneity, each of them can result in huge deformations and bias [12]. Economic development, quick policy modifications, political disagreements, and financial crises all contribute to regime change, adding asymmetry to the GF-GTI relationship [4]. Consequently, this investigation will examine every economy solely to tackle the aforementioned concerns. Fourth, irrespective of their near locality, these countries are typically quite autonomous since each holds distinctive features in terms of GF effectiveness in improving GTI [14]. As a consequence, to cope with country-specific heterogeneity, an econometric instrument in particular QQ is necessary for empirical model building. We expect that the results of the investigation will give a more extensive picture of the linkage between the aforementioned elements that might be complicated to get utilizing typical methodologies. Finally, the findings of this empirical work would offer new ways for further probe into the GF-GTI link and its relevance for different nations.

The remaining portions of the investigation are categorized as follows: Section 2 offers the theoretical framework of the research. Section 3 gives a summary of past empirical works. The fourth section of the study examines the data, and the fifth section outlines the econometric approach. Section 6 includes initial and key outcomes of the study, coupled with an exhaustive discussion. In the end, section 7 ends with summarized outcomes along with policy suggestions.

## 2 Theoretical framework

GTI is directly influenced by GF, mostly through financial assistance, information sharing, allocation of resources, and risk mitigation. Regarding financial assistance, GF may extend financing channels for green innovation issues while lowering financing costs [14]. GTI incorporates a significant digit of tiny and moderate-sized businesses that typically face high interest rates and financing challenges due to a shortage of assets and capital. GF promotes the policy of green innovation by encouraging the use of GB, green funds, green insurance, and green credit [15]. It effectively expands the green industry financing methods to build a broad policy of investment themes with the involvement of foreign capital, private capital, financial intermediaries, government funds, and pension funds. Meanwhile, by optimizing the financial framework, GF lowers the financing expenses of green industries while increasing polluting projects to enable more businesses to participate in GTI. From the information sharing perspective, GF may minimize resource-matched costs by encouraging collaboration and knowledge exchange among firms, financial institutions and enterprises. Simultaneously, the government is actively involved in modifying current financial instruments to widen financing channels for the development of GTI and current fiscal policy through revenue and distribution adjustments [16].

As for resource allocation, GF may enhance GTI by promoting green investment, inhibiting polluting investment, and fostering capital orientations [17]. Conversely, financial institutions can limit the total amount of funds accessible to businesses by limiting further loans to vastly polluting firms and restricting the overall size of funds to reduce polluted investments and incentivize firms to adopt GTI [18]. Alternatively, GF may stimulate the growth of the green energy industry through the usage of financial subsidies, equity investments, green loans, and GTI. Furthermore, GF may drive social capital to participate in the renewables industries while also recruiting additional land, social capital, labor, and other essential resources for the green sector [17,18].

As for risk management, GF can effectively diversify GTI risks and boost organizations' capabilities and innovation capabilities. By harnessing the inherent benefits of risk redistribution [19], GF may alleviate knowledge asymmetries and market failures and effectively disperse risk across all dimensions of GTI. Green insurance, for instance, can compensate for damages experienced by green firms during the manufacturing and innovation processes [20]. Furthermore, completing green financial assessment and integrating the outcomes into the quantitative evaluation process would encourage financial institutions to be additional uniform in relating to corporate governance, risk assessment, and corporate development while also encouraging financial institutions to maximize the allocation of their green assets and boost their incentives for green financial innovations and their capacity to handle environment degradation. From the standpoint of GTI indicators, first and foremost, implementing GTI initiatives by businesses may strengthen their capabilities and hence provide competitive advantages [21]. Second, the reduction of institutional burden is a strong motivator for businesses to implement environmental protection strategies [22]. Furthermore, the government's continuous growth in environmental regulations will effectively drive businesses to implement novel environmental solutions. Finally, pressure from stakeholders like customers and suppliers will motivate businesses to enhance their environmental performance [23].

## 3 Literature review

With the rise of environmental issues in current years, the issues of GTI and GF have gained considerable attention. Multiple studies have revealed the impact of GF on diverse macroeconomic indicators ([24]; [25] [16,26]; Tiwari et al., 2022). Though, comparatively few studies have investigated the GF-GTI link [6,4,10,27].

[3] evaluated the role of GF in GTI in China. Fixed effect model was used by taking the data of 30 regions of China from 2008 to 2019. It was found that GF significantly and positively enhanced GTI in all regions of China. Owen et al. [28] scrutinized the influence of GF on GTI by examining the requirements for longer-term investments in GTI activities and concluded that authorities should implement policies to encourage greater funding in initial-stage GTI. In the same way, Liu, Wang, and Cai [29] stated that GF is critical to assisting environmentally friendly businesses in carrying out green initiatives and procedures. Tang & Zhang [30] also noted that GB benefits shareholders by encouraging their companies to pursue green-related industries, which may eventually stimulate GTI. According to Kudratova et al. [6], green ventures provide higher gains for investors as compared to conventional innovation, so green funding may stimulate renewable energy R&D. Li et al. [7] maintained the notion that green bank credits are useful in promoting GTI by performing a hypothetical study among banks, governments, and enterprises based on the green loan theory. Yu et al. [27] found that GF improved enterprises' GTI advancement by overcoming financing limitations by using data from Chinese-listed firms for the years 2001–2018. Furthermore, privately held companies' greening capacities are more subject to finance limitations than economy-owned organizations. Yaoteng & Xin [10] used a spatial model to observe the impact of GF over GTI and argued that GF helps GTI. Wang et al. [1] probed the effect of environmental impact and GF on GTI. The panel data of 57 developing countries were used for the years 2002–2016. The results of PMG estimation suggested that environmental performance has a positive association with GTI in the non-emerging nations and the economies with better GTI. Furthermore, GF has a positive relationship with GTI in emerging economies and economies with lower GF levels. Moreover, GF negatively affected GTI in the economies with better GTI.

Huang et al. [2] explored the GF-GTI nexus by taking the panel dataset regarding 30 Chinese states from 2008 to 2019. The results of spatial Durbin and panel threshold models have examined that GF and GTI had a positive significant correlation. The indirect and direct influence of GF on GTI was significantly positive. On the other hand, double threshold impacts of GF on GTI showed that the driving influence of GF was decreased by increasing the intensity of environmental regulations. Furthermore, Li & Jia (2017) discovered that ecological finance was the most efficient technique for mitigating environmental degradation. GF boosts investment in fresh innovations and ideas like renewable energy. Hong et al. [4] evaluated the influence of green loan strategies over corporate GTI by taking the panel data of 2826 listed firms in China for the years 2007–2019. The estimates of the Difference-in-Difference model identified that green credit guidelines promoted corporate GTI as a whole. Green credit norms reduced GTI mostly through debt reduction rather than funding limits. Furthermore, the influence of green credit guidelines on GTI was heterogeneous. Green credit procedures indicated a significant and beneficial influence on the GTI of public sector firms but showed no influence on the GTI of non-country-owned firms.

We can see from the preceding literature that most work stressed the entire impact of GF on GTI and scarcely probed the relationship between various quantiles of GF and GTI. No study has been found about GF-GTI relations within the context of European countries. Moreover, the former investigations studies assume a linear association between the variables. Accordingly, failing to consider non-linear effects could result in information loss and erroneous findings. Further, earlier research has tended to concentrate on panel data in place of time-series data. Panel data analysis exhibits a number of obstacles, like the issue of generalization, misuse of measurements, and poor choice of model. The variation in the allocation of GF in panel data forms it impractical to estimate the mean impacts of environmental assistance in terms of GTI for every economy. Resultantly, it is encouraged to use QQ, a powerful econometric method, to examine the influence of GF on GTI for each nation independently.

## 4 Data and description

Our work appraises the connection between GF and GTI for the ten EU nations that promote GF (Germany, Sweden, France, Italy, Netherlands, Spain, Denmark, Norway, Belgium, and Finland). The data set contains two variables. We adopt GF as the independent variable, which is estimated by the GB calculated through the S&P green bond index [31]. We have acquired the quarterly series of GB data from DataStream. GTI is the dependent variable. We acquire annual GTI data from OECD-Statistics,^1^ which is subsequently converted into quarterly data by employing the method of quadratic match-sum, chased by Sharif et al. [11]. By fitting point-to-point discrepancies, this methodology balances data for occasional cycles while transitioning from higher to lower frequencies [11,32]. With 133 monthly observations, the data spans from December 2008 to December 2019 (for both GF and GTI). The taxonomy of the symbols and acronyms chosen in the article is listed in Table 1 in the starting of this paper.

**Table 1:** The list of symbols and acronyms.

Symbol or Acronym	Narration	Symbol or Acronym	Narration
GF	Green finance	τ	τ^th^ quantile of green technology innovation
GTI	Green technology innovation	μ_t_^σ^	Quantile error term
GB	Green bonds	h	parameter for bandwidth
QQ	Quantile-on-Quantile Estimation	$\rho_{\varphi }$	quantile loss function
ADF	Augmented Dickey-Fuller	Sup_τ_ |V_n_(τ)|	Supremum norm value for the coefficients (β and δ)
J-B	Jarque-Bera test	s	Number of quantiles
QR	Quantile regression	EU	European Union

## 5 Econometric technique

The ongoing section elucidates the econometric tool employed in the present work. To explore the long run linkage throughout the variables, a quantile-based cointegration test is executed. Furthermore, we adopt a QQ tool for econometric estimation.

### 5.1 Quantile cointegration (QC) test

Occasional failures in detecting long-run correlations between variables may be attributed to the utilization of persistent cointegration vectors in customary cointegration tests [33]. We opt for the quantile cointegration test of Xiao [33] to remove estimate bias. The QC test incorporates temporal oscillations jointly with the linkage throughout various quantiles of independent and dependent variables to evaluate long-run correlations in conditionally distributed data. Due to traditional cointegration tests that have endogeneity restrictions, Xiao [33] updated them to confront the necessities of Saikkonen [34] by containing fragment cointegration errors within lead-lag elements.

The persistent vector α(τ) is shown in Equation (1), according to which the cointegration model might be represented as follows:

(1) $$X_{i}=\alpha +\alpha^{\prime }Y_{i}+\sum_{k=-s}^{k}\Delta Y_{i-k}^{\prime }\Pi_{k}+v_{i}$$

and

(2) $$Q_{\tau }^{X}\left(X_{i}{N_{i}}^{X},{N_{i}}^{y}\right)=\beta \left(\tau \right)+\alpha {\left(\tau \right)}^{\prime }Y_{i}+\sum_{k=-s}^{s}\Delta Y_{i-k}^{\prime }\Pi_{j}+F_{v}^{-1}\left(\tau \right)$$

In Equation (2), β(τ) is a drift factor that displays constant parameters. When considering the conditional data distribution, the quantile errors are delineated by the symbol $F_{v}^{-1}\left(\tau \right)$. The quadratic term of the cointegration would be articulated as under:

(3) $$Q_{\tau }^{X}\left(X_{i}{N_{i}}^{X},{N_{i}}^{y}\right)=\beta \left(\tau \right)+\alpha {\left(\tau \right)}^{\prime }Y_{i}+\delta {\left(\tau \right)}^{\prime }{Y_{i}}^{2}+\sum_{k=-s}^{s}\Delta Y_{i-k}^{\prime }\Pi_{k}+\sum_{k=-s}^{s}\Delta Y_{i-k}^{2}\Pi_{k}+F_{v}^{-1}\left(\tau \right)$$

The coefficients of cointegration for the QC test are obtained using H_0_: α (π) = α as the null hypothesis, which is extracted from Equation (3). In our research, ${\hat{V}}_{n}\left(\tau \right)=\left[\hat{\alpha }\left(\tau \right)-\hat{\alpha }\right]$ stand for the null hypothesis (supremum rule). In current work, $Sup_{\tau }\left|V_{n}^{\prime }\left(\tau \right)\right|$ is adopted as a test statistic used for complete quantile distributions. The crucial values of the test statistic are identified by adopting a method that involves conducting 1000 Monte Carlo simulations.

### 5.2 Quantile-on-quantile (QQ) approach

In view of our investigation, we utilize the QQ tool as the preferred methodology for creating a connection between the dual variables based on their asymmetric distribution. The ordinary quantile regression (QR) model solely scrutinizes the average provisional impact of the independent variable on various dependent variable quantiles. The QQ approach is originated by Sim & Zhou [35] as a solution to resolve the limitations present in the existing QR method. The QQ technique proves advantageous in evaluating various dimensions of the linkage between quantiles of both independent and dependent variables [35]. This approach integrates non-parametrical estimates with traditional QR, enabling the evaluation of how the independent variable's quantile influences the dependent variable's quantile, therefore addressing the issue of interconnection. Therefore, the QQ technique is being used in ongoing study to disclose challenges in the GF-GTI that may be elusive when utilizing other typical econometric tools, including QR and OLS.

In this study, we employ a simplified version of the non-parametric model, supported by the research of Tang & Zhang [30] and Hong et al. [4], in the following manner:

(4) $$GTI_{t}=\alpha^{\sigma }\left(GF_{t}\right)+{\mu_{t}}^{\sigma }$$

In Equation (4), GF and GTI denote green finance and green technology innovation, respectively, in the t time period. σ exhibits the σ^th^ quantile of the provisional GTI distribution. Given the lack of earlier information regarding the bond between GF and GTI, it is logical to expect that the load factor α^σ^(.) is unfamiliar. The σ^th^ quantile is illustrated by the quantile error term indicated by ${\mu_{t}}^{\sigma }$.

To appraise Equation (4) in the vicinity of TUN, we utilize local linear regression in the following manner:

(5) $$\alpha^{\sigma }\left(GF_{t}\right)\approx \alpha^{\sigma }\left(GF^{\tau }\right)+\alpha^{\sigma^{\prime }}\left(GF^{\tau }\right)\left(GF_{t}-GF^{\tau }\right)$$

The partial influence or derivative of α^σ^ (GF_t_) concerning GFt is designated as α^σʹ^. The signs σ and τ stands forα^σ^(GF^τ^) and α^σ^(GF^τ^) correspondingly. α_1_(σ,τ) symbolizes α^σʹ^(GFτ), while α_0_(σ,τ) represents α_σ_(GFτ). Consequently, an expanded form of Equation (5) can be stated as under:

(6) $$\alpha^{\sigma }\left(GF_{t}\right)\approx \alpha_{0}\left(\sigma ,\tau \right)+\alpha_{1}\left(\sigma ,\tau \right)\left(GF_{t}-GF^{\tau }\right)$$

We present the further QQ regression by placing Equation (6) in Equation (4):

(7) $$GTI_{t}=\frac{\alpha_{0}\left(\sigma ,\tau \right)+\alpha_{1}\left(\sigma ,\tau \right)\;\left(GF_{t}-\;{\text{GF}}^{\tau }\right)\;}{\left(*\right)}+u_{t}^{\sigma }$$

The empirical form of the QQ model is shown in Equation (7), which explains the alliance between the σ^th^ GF quantiles and the τ^th^ GTI quantiles. The conditional quantiles of GF are denoted by the term (*). The connection between the quantiles of GF and GTI is established through the doubly indexed parameters α_0_ and α_1_, which vary according to the GF-GTI quantiles. The parameters α_0_ and α_0_ exhibit variability in accordance with the values of σ and τ. Equation (7) unveils the intrinsic GF-GTI relationship by merging their respective distributions, thereby exposing the underlying dependency pattern. In terms of performance, the QQ method beats other commonly used time-series methods, irrespective of being a bivariate approach that only utilizes a single independent variable [12]. By accounting for the linkage between GF and GTI across each of the upper and lower quantiles, the QQ method can anticipate and deliver more precise and reliable outcomes compared to other usually adopted approaches [13].

The significance of bandwidth in the minimization problem lies in its indication of the linkage between the quantiles of GTI and GF.

(8) $$Mi{n_{\delta_{0}}}_{\delta_{1}}\sum_{t=1}^{n}\rho_{\varphi }\left[GTI_{t}-\delta_{0}-\delta_{1}\left(GF_{t}-GF^{\tau }\right)\right]L\left[\frac{M_{n}\left(GF_{t}\right)-\tau }{h}\right]$$

In Equation (8), $\rho_{\varphi }$ represents quantile regression loss function, which measures the accuracy of a prediction. We use a function L (.) called the Gaussian kernel to give different weights to data points that are closer or farther away from a certain point. The Gaussian kernel works by comparing the distribution of the data around that point with a standard distribution. We calculate the ratio between the observed distribution and the expected distribution based on certain GF quantiles. This ratio helps us determine the weights for each data point. In this context, 'h' represents a parameter called bandwidth, which controls how wide or narrow the distribution is. 'I' is an indicator function that tells us whether a data point is inside a certain range or not. Bandwidth works as a smoothing constituent in kernel regression, lessening both variability and bias. Estimations procured with a narrow bandwidth result in higher variance, while estimates obtained with a wide bandwidth tend to exhibit a more skewed distribution [35]. Thereby, achieving an optimal balance between sway and variance is crucial. A bandwidth limit of h = 0.05 is adopted for this research, in line with previous research of Sinha, Mishra, Sharif, & Yarovaya [36].

### 5.3 Robustness of the QQ technique

The QQ regression allows us to obtain conventional QR measures by facilitating accurate evaluations for multiple quantiles of GF. While traditional QR estimates are only based on a single parameter (σ), it can estimate the effect of the σ^th^ GF quantile (independent variable) on GTI (dependent variable). Contrarily, QQ analysis considers the linkage between σ^th^ GF quantile and the τ^th^ GTI quantile, leading to detailed data analysis. By averaging the QQ parameters across τ, we derive the slope coefficients for the QR procedure. These slope coefficients, symbolized as $\delta_{1}\left(\sigma \right)$ , are then implemented to ascertain the effect of GF on numerous GTI quantiles, as shown under.

(9) $$\delta_{1}\left(\sigma \right)\equiv {\bar{\hat{\alpha }}}_{1}=\frac{1}{s}\sum_{\tau }{\hat{\alpha }}_{1}\left(\sigma ,\tau \right)$$

In the given situation, we have 19 quantiles represented by the variable ‘s’ in Equation (9). The range of quantiles is designated by τ, which includes values like 0.05, 0.10, 0.15, and so on, up to 0.95. To estimate the vitality of the QQ regression, we match the determined QR parameters with the averaged QQ estimations across the range of quantiles. This allows us to determine how consistent and trustworthy the QQ technique provides accurate results.

## 6 Findings and discussion

This section contains the initial and major outcomes of the article, coupled with an extensive illustration of the findings.

### 6.1 Preliminary findings

The descriptive stat values of GTI and GF are presented in Table 2.

**Table 2:** Descriptive statistics values for GF and GTI.

Countries	Mean	Max.	Min.	Std. Dev.	J-B Stats	ADF Level	ADFΔ
Panel A: Green Finance (GF)							
Germany	124.28	148.45	93.08	11.16	54.27*	−1.48	−4.62*
France	123.86	157.23	91.56	17.17	261.57*	−0.93	−6.76*
Sweden	122.40	140.24	90.64	11.46	406.12*	−1.42	−4.31*
Netherlands	120.40	134.41	90.70	7.77	201.98*	−1.52	−5.61*
Italy	117.74	149.46	91.73	17.95	326.28*	−1.34	−5.85*
Spain	112.90	141.12	96.47	11.47	253.30*	−1.76	−5.82*
Denmark	111.60	133.21	88.04	11.41	243.80*	−1.68	−5.03*
Norway	95.80	104.56	77.89	3.20	396.70*	−1.69	−4.81**
Belgium	92.51	113.95	75.57	10.01	237.81*	−1.97	−5.78*
Finland	89.74	108.17	81.70	4.69	324.42*	−2.15*	−4.50**
**Panel B: Green Technology Innovation (GTI)**							
Germany	154.48	197.82	132.63	17.59	21.26*	−1.70	−5.65*
France	1109.70	1262.76	850.76	112.41	8.37*	−4.32*	−6.77*
Sweden	405.35	487.76	242.67	53.01	25.32*	−1.72	−4.46*
Netherlands	356.38	408.65	293.88	24.29	2.09*	−1.56	−5.76*
Italy	373.87	415.76	253.76	27.54	144.17	−5.72*	−5.25*
Spain	247.76	303.87	157.76	40.51	1.16	−1.84	−4.13*
Denmark	273.72	351.76	214.27	46.78	14.75*	−1.89	−3.73**
Norway	114.11	150.72	50.34	20.01	11.02*	−1.10	−5.75*
Belgium	154.48	197.65	132.63	17.69	21.26*	−1.66	−5.74*
Finland	208.83	271.76	163.76	31.05	10.26*	−1.86	−5.18*

Note: * and ** depict the level of significance at 1 ^%^ and 5 ^%^, correspondingly.

With a mean value of GB prices as 124.28, ranging from 93.08 to 148.45, Germany is the top GF supporter country. France holds the second rank containing an average score of GF (123.86), with values ranging from 91.56 to 157.23. Sweden is third, followed by the Netherlands, Italy, and Spain. France contains the topmost rank of GTI, with a mean value of 1109.70, ranging from 850.76 to 1262. Sweden ranks second, having an average value of GTI 405.35, extending from 242.67 to 487.76. Italy claims the third rank, followed by Netherlands, Denmark, and Finland. The JB test outcomes demonstrate that GF and GTI have non-normal data distribution in our sampled economies, excluding Spain, where GTI shows normal distribution. Moreover, our countries' non-normal data distribution strengthens the rationale of the QQ methodology that is ideal in such cases [11]. In most of selected economies, the ADF test exhibits that the variables are stationary at their first differences. Consequently, we exerted a stationary series of data by modifying all our variables into their first differences, pursued by the work of Shahbaz et al. (2018).

Table 3 shows correlation coefficients of GF and GTI. Except for Italy, GF and GTI are positively and highly linked with each other as per correlation coefficients for entire economies. The topmost correlation coefficient (0.86) is detected in Germany, followed by Norway (0.85), Denmark (0.80), and Finland (0.76).

**Table 3:** Correlation between GTI and GF.

Nation	Correlation	t-Stats	p-value
Germany	0.86	10.78*	0.000
France	0.69	7.60*	0.000
Sweden	0.74	4.04*	0.000
Netherlands	0.65	7.80*	0.000
Italy	−0.55	−2.81*	0.000
Spain	0.75	7.39*	0.000
Denmark	0.80	26.35*	0.000
Norway	0.85	11.27*	0.000
Belgium	0.74	4.09*	0.000
Finland	0.76	5.35*	0.000

Note:’*’ shows the significance level at 1 ^%^.

### 6.2 Major outcomes

The outcomes attained by the QC test are indicated in Table 4. τ depicts the τ^th^ GTI quantile. The coefficients of the supremum norm (β and δ) express the parameters' consistency.

**Table 4:** Results of quantile cointegration (QC) test.

Economy	Coefficient	Sup_τ_ |V_n_(τ)|	CV (1 %)	CV (5 %)	CV (10 %)
Germany	β	8318.20	5280.20	3139.06	2535.35
GF vs. GTI	δ	177.60	106.40	51.85	38.30
France	β	3931.90	3766.20	248.54	202.95
GF vs. GTI	δ	160.77	159.85	48.08	46.77
Sweden	β	1835.70	1540.79	1041.70	997.80
GF vs. GTI	δ	945.70	686.62	490.74	345.86
Netherlands	β	6238.60	5680.90	4688.50	3788.70
GF vs. GTI	δ	3272.90	2693.77	2185.70	1859.41
Italy GF vs. GTI	β	68590.31	58350.30	57318.20	54881.75
	δ	2456.60	1498.20	1438.41	1430.18
Spain	β	1244.78	939.70	545.09	205.70
GF vs. GTI	δ	784.80	586.90	497.90	376.73
Denmark	β	9380.06	7204.07	5908.70	2623.08
GF vs. GTI	δ	253.60	165.45	128.58	98.15
Norway GF vs. GTI	β	8753.50	6710.10	4770.10	1475.80
	δ	397.10	203.60	102.08	98.10
Belgium	β	538.40	325.35	294.70	237.57
GF vs. GTI	δ	288.90	198.80	125.70	98.30
Finland	β	7116.35	3495.13	3085.18	2228.30
GF vs. GTI	δ	605.40	301.85	216.16	118.12

**Note:** To estimate the t-stat values of the QC test, a grid of 19 smoothly-spaced quantiles extending from 0.05 to 0.95 is adopted. The estimates of the parameters (β and δ) using the supremum norm, along with their critical values at the levels of significance of 1 %, 5 %, and 10 %, are given as CV(1 %), CV(5 %), and CV(10 %), correspondingly.

The findings collected by the QC test express that the long-run GF-GTI bond alters by quantiles for every nation. The coefficients hold high supremum norm scores relative to analogous critical limits (CV1%, CV5%, and CV10 %), establishing that GF and GTI have a long-run, non-linear, and significant association in selected EU nations.

**Fig. 1:**
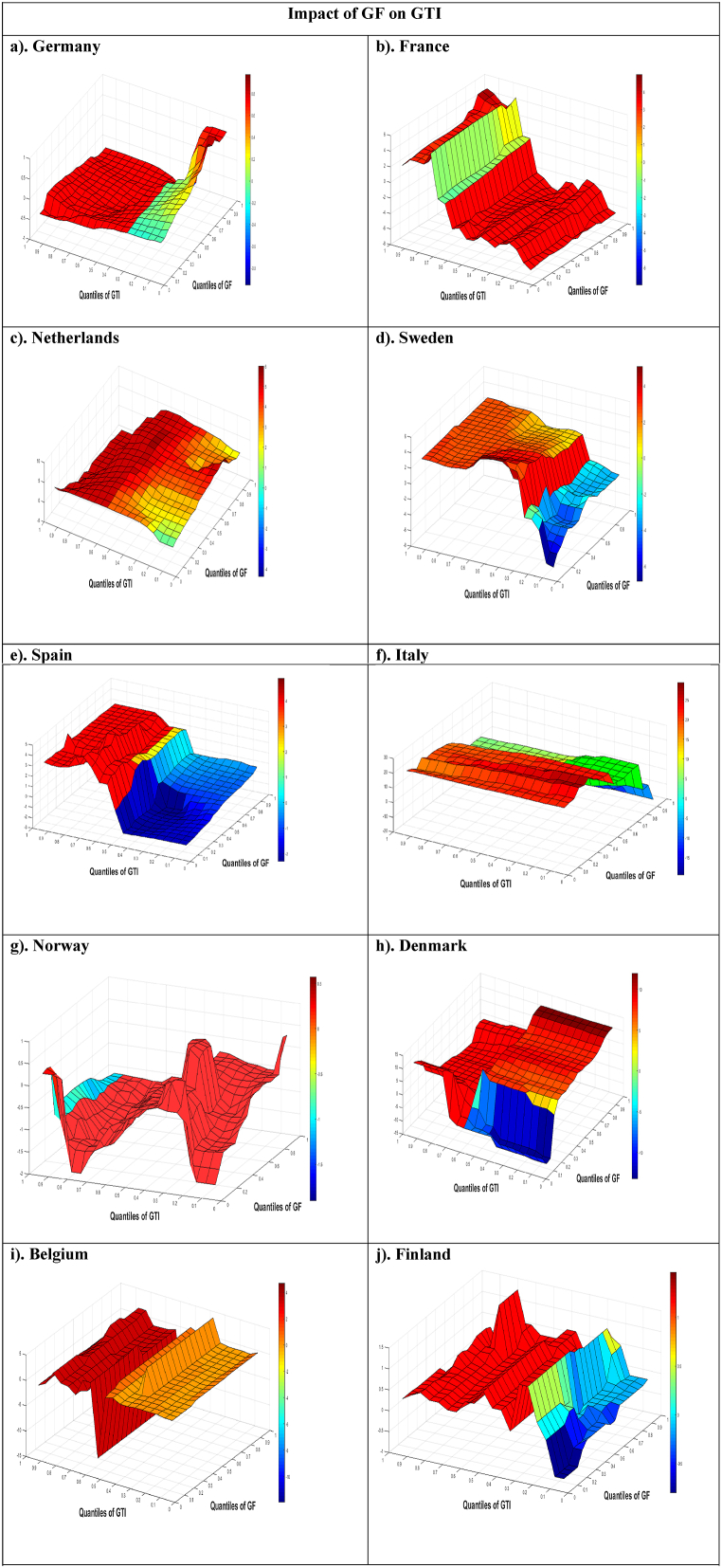
**QQ Estimations of the Slope Coefficient** α_1_ (σ, ***τ***) Note: The slope coefficients α_1_(σ, ***τ***) are displayed on the z-axis, while the GF quantiles are represented on the x-axis, and the GTI quantiles are represented on the y-axis in the presentation of the estimations.

Fig. 1 exhibits the estimations of slopes α_1_(**σ**, ***τ***) that expose the effect of the **σ**^**th**^ GF quantile on the ***τ***^th^ GTI quantile in multiple countries adopting diverse values of **σ** and ***τ***. In **Germany** and the **Netherlands,** a positive and powerful influence of GF on GTI is preeminent as displayed in Fig. 1(a) and (c), respectively. The positive strong correlation among the GF-GTI quantiles is observed across portions, which add complete GF quantiles associating the lower-medium to topmost GTI quantiles in both economies. This exclusively powerful positive bond entails that the GF expands GTI at rising levels of innovations. The results are assisted by the investigation of Hong et al. [4] and Kudratova et al. [6], who perceived that GF raises GTI. However, a weak positive GF-GTI link is similarly viewed among points, which integrate GF quantiles associating bottommost to mid-low GTI quantiles (0.05–0.35). It means GF has an insignificant or weak bond with GTI when GTI is at its low levels. In **France,** a positive powerful impact of GF on GTI is prominent (as shown in Fig. 1(b). A powerful direct GF-GTI bond is seen across places, which join overall quantiles of GF, associating lowest to moderate-higher and top GTI quantiles (0.05–0.65 & 0.85–0.95). The significant positive link implies that the GF significantly increases GTI at lowest to median and upper innovation levels. The findings are confirming the research of Yu et al. [27] and Yaoteng & Xin [10], who also observed a positive GF-GTI association. Though, a weak negative GF-GTI link is too realized in the segments that connect complete GF quantiles with higher-mid GTI quantiles (0.70–0.80).

**Sweden** in Fig. 1(d) and **Finland** in Fig. 1(j) show a positive and vigorous impact of GF on GTI. The vigorous positive linkage among the GF-GTI quantiles is established among domains, which nexus complete GF quantiles and the lower-medium to highest GTI quantiles (0.40–0.95). The thoroughly paramount positive tie entails that the GF significantly improves green technological advancements at rising levels of GTI. However, an inverse powerful GF-GTI tie is also witnessed within constituencies, which link entire quantiles of GF associating bottom to moderate-low GTI quantiles. This essentially negative powerful relationship necessitates that the GF reduces GTI in Sweden and Finland at lower levels of innovations. **Norway** in Fig. 1(g) shows the powerful and positive influence of GF on GTI. The positive powerful GF-GTI nexus is detected all over divisions, which tie entire GF quintiles associating lowest to high-mid GTI quantiles. This particularly powerful positive relationship implies that the GF significantly boosts GTI at lower to top GTI levels. The positive GF-GTI connection is affirmed by the works of [3] and Liu et al. [29]. However, an inverse weak GF-GTI relationship is also initiated within vicinities that combine whole GF quantiles, associating uppermost GTI quantiles (0.90–0.95). This situation shows that GF has an insignificant impact on GTI in Norway when GTI is at its peak.

In Fig. 1(h), **Denmark** designates a positive powerful effect of GF on GTI. An extremely positive GF-GTI linkage is witnessed throughout sections that interrelate moderately lower to upmost GF quantiles (0.45–0.95), associating moderately high to topmost GTI quantiles (0.60–0.95). The essentially powerful positive relationship necessitates that GF expands GTI at vigorous ranks of GF. However, a powerful negative GF-GTI association is also witnessed in the localities that tie lowest to low-mid GF quantiles (0.05–0.40), associating bottommost to median GTI quantiles (0.05–0.55). In Fig. 1(i), **Belgium** reveals a positive powerful influence of GF on GTI. The vigorous positive GF-GTI interconnection is found throughout sections, which merge complete GF quantiles associating low-median to uppermost GTI quantiles (0.45–0.95). The expressly positive robust interrelation discloses that the GF substantially upgrades GTI at moderate to higher ranks of GTI in Belgium. The positive association between GF and GTI in our analysis is backed by the works of Tang & Zhang [30] and Yu et al. [27]. However, a weak positive GF-GTI tie is also presented within areas that associate complete GF quantiles relating lowermost to moderately low GTI quantiles (0.05–0.40).

Contrary to the above estimates, Fig. 1(e) shows that **Spain** depicts a mixed GF-GTI correlation. A positive significant GF-GTI interrelation is assessed throughout points, which link complete quantiles of GF associating high-medium to upper GTI quantiles (0.55–0.95). The exceptionally vigorous positive bond implies that the GF increases GTI in Spain at moderate to higher ranks of GF. Though, a strong negative GF-GTI relationship is too witnessed within points, which tie complete GF quantiles linking lowest to mid GTI quantiles (0.05–0.50). It depicts that GF increases the levels of GTI at the time of low to median GF levels. Like Spain, **Italy** also shows mixed results in Fig. 1(f). The vigorous positive GF-GTI union is evident throughout localities, which integrate lowest to median GF quantiles (0.05–0.50) associating overall GTI quantiles. The particularly positive powerful relationship implies that the GF boosts GTI at times of bearish to moderate GF market conditions in Italy. Moreover, a strong negative GF-GTI nexus is observed in the portions that tie mid-higher to foremost quantiles of GF (0.75–0.95) and entire GTI quantiles. The extremely vigorous negative interconnection asserts that the GF reduces GTI at times of bullish GF market situation. However, a feeble inverse GF-GTI alliance is witnessed throughout the locations combining medium-high GF quantiles (0.55–0.70) and whole GTI quantiles. The mixed GF-GTI connection in Spain and Italy is assisted by Wang et al. [1], who also found both negative and positive effects of GF on GTI.

Table 5 exhibits a brief outline of the linkages throughout multiple quantiles of GF and GTI for our EU economies. We find a positive and significant GF-GTI connection in almost all nations, denoting that GF boosts GTI. Nonetheless, there exists a mixed associations between GF and GTI in Italy and Spain.

**Table 5:** Summary of Findings (Correlation b/w Different Quantiles of GF and GTI).

Economies	GF Quantiles	GTI Quantiles	Relationship b/w Quantiles	Dominant Relationship
Germany	Whole quantile**s**	Low-mid to topmost quantile**s**	Strong positive	Positive powerful
	Complete quantile**s**	Bottommost to low-mid quantiles	Feeble positive	
France	Whole quantiles	Bottom to high-medium and topmost quantiles	Positive strong	Strong positive
	All quantiles	Moderately higher quantiles	Fragile negative	
Netherlands	Entire quantiles	Mid-low to higher quantiles	Powerful positive	Positive strong
	Overall quantiles	Bottom to Lower-middle quantiles	Positive fragile	
Sweden	Whole quantiles	Medium-lower to high quantiles	Positive powerful	Powerful positive
	Overall quantiles	Lowermost to lower-middle quantiles	Powerful negative	
Spain	Entire quantiles	Medium-high to top quantile**s**	Strong positive	Mixed relationship
	Complete quantile**s**	Lowest to median quantiles	Negative strong	
Italy	Low to middle quantile**s**	Overall quantiles	Powerful positive	Mixed relationship
	Medium-upper to highest quantile**s**	Entire quantiles	Inverse strong	
	Moderately high quantiles	Whole quantiles	Fragile negative	
Norway	Whole quantiles	Lowest to high-mid quantiles	Positive strong	Strong positive
	Complete quantiles	Highest quantile**s**	Feeble negative	
Denmark	Moderately low to top quantile**s**	Higher-mid to top quantile**s**	Powerful positive	Powerful positive
	Bottommost to low-middle quantile**s**	Moderately low quantiles	Strong negative	
Belgium	All quantiles	Moderate-low to higher quantiles	Positive powerful	Powerful positive
	Whole quantiles	Lowest to moderately low quantiles	Feeble positive	
Finland	Entire quantile**s**	Low-mid to higher quantiles	Positive strong	Positive powerful
	Complete quantile**s**	Bottom to lower-middle quantile**s**	Powerful negative	

### 6.3 Affirming the robustness of the QQ methodology

**Fig. 2:**
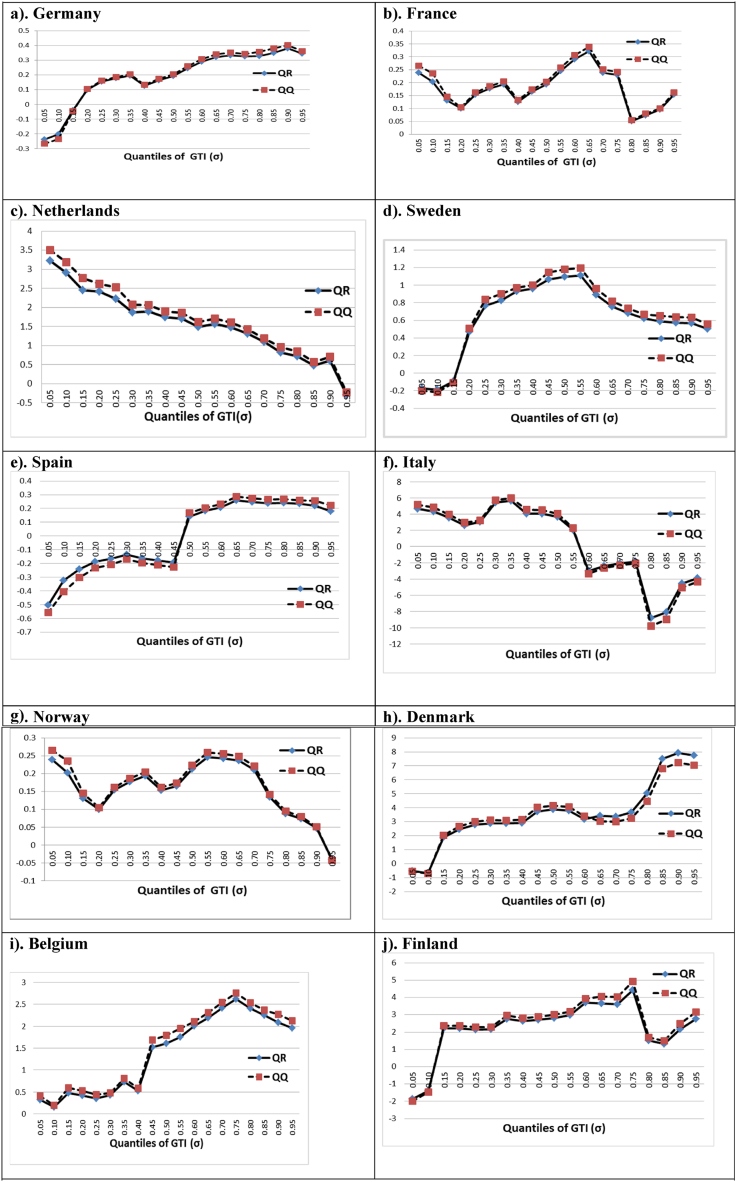
Verifying the Robustness of the QQ Method by Matching QQ and QR Tools Note: The estimates of the standard QR parameters and the average parameters of QQ are presented against various GTI quantiles.

We now establish a bond between the QQ and QR estimations to determine their level of agreement. Fig. 2 affirms the former QQ estimates. As per the figures, the averaged-QQ estimates of slope coefficients follow an identical behaviour to the QR estimates.

Fig. 2 demonstrates a positive GF-GTI connection in most economies like France in Fig. 2(b), Netherlands in Fig. 2(c), Germany in Fig. 2(a), Sweden in Fig. 2(d), Finland in Fig. 2(j), Belgium in Fig. 2(i), Norway in Fig. 2(g), and Denmark in Fig. 2(h). Furthermore, Spain in Fig. 2(e) and Italy in Fig. 2(f) have mixed findings. Furthermore, our results show that GF and GTI levels fluctuate throughout all nations. The coefficients’ ranges interpret that the influence of GF on GTI is much bigger in Finland, Denmark, and Italy but relatively lower in Norway and France.

### 6.4 Discussion of findings

The findings exhibit a positive GF-GTI connection in multiple countries. GF has been detected to be a reason for expanding GTI in 8 out of 10 selected nations, as have other empirical works, like Tang & Zhang [30] and Yu et al. [27], that entail that GF boosts GTI. Our outcomes of a positive GF-GTI relationship are somewhat consistent with those of [3] and Yu et al. [27] for China, Wang et al. (2022) for developing economies, and Tolliver, Fujii, Keeley, & Managi [25] for Asian countries, who argue that GF enhances GTI.

The impact of GF and GTI in Spain and Italy exhibits mixed outcomes across various quantiles. This observation is sustained by Wang et al. [1], who conducted a study revealing a time-fluctuating union between GF and GTI. They argue that the influence of GF on GTI could not consistently result in a higher GTI due to elements containing locality, time, and cyclical fluctuations. Furthermore, the effect of increased GF on GTI might be detrimental, depending on the corporate ownership situation in a country [1]. The mixed association between GF and GTI across different quantiles and economies can be attributed to several reasons. First, the GF effect is likely to vary depending on the specific quantiles examined. Different quantiles represent distinct segments of the data distribution, which may encompass varying levels of environmental sustainability and counterterrorism efforts. Consequently, the relationship between GF and GTI could differ significantly across these quantiles. Second, economies such as Spain and Italy, possess unique characteristics and circumstances that can contribute to the diverse outcomes observed. Factors such as geographical location, economic structure, political environment, and cultural context may interact with the influence of GF on GTI, leading to mixed results. These contextual factors can shape the effectiveness of GF initiatives and their impact on counterterrorism efforts differently in each country. Moreover, the study by Wang et al. [1] suggests that the junction between GF and GTI is influenced by cyclical fluctuations. This implies that the effectiveness of GF in reducing terrorism may vary over time, depending on the prevailing economic conditions, social dynamics, and political stability. As a result, the impact of GF on GTI may not be consistent or linear, further contributing to the mixed outcomes observed across quantiles and economies. A multitude of factors, comprising green investment behavior, risk perception, green energy consumption, technological development, and business cycles, might explain this mixed association between GF and GTI. The mixed GF-GTI connection is also assisted by Hong et al. [4], who discovered that the GF-GTI association shows heterogeneous behavior. Moreover, the results of Spain are supported by Zho, Tang, & Zhang (2020), who contend that the GF level is helpful to the environment just later a particular time, before which GF is not beneficial. It is evident that the higher quantiles of GTI imply a stronger link with GF in most of our selected EU nations (i.e., Sweden, France, Netherlands, Germany, Spain, Denmark, Finland, and Belgium). It implies that when GTI grades are higher, GF significantly enhances GTI by increasing technological advancements. This observation suggests that when there is a greater focus on developing and implementing green technologies, the availability and accessibility of GF play a significant role in fostering technological advancements in the green sector. By providing financial resources and incentives, GF facilitates the development, research, and adoption of green technologies. These financial resources can be used to fund research and development, improve infrastructure, promote sustainable manufacturing practices, and incentivize companies to adopt and scale up green innovations. Furthermore, the positive relationship between GTI and GF in the selected EU nations suggests that when GTI levels are high, the impact of GF becomes more pronounced. This can be attributed to the fact that with a powerful foundation of GTI, there is a greater potential for technological advancements and scalability. GF can then act as a catalyst, further accelerating the progress by providing the required financial support for the research, development, and commercialization of these technologies.

As previously indicated, discrepancies in the effect of GF between sampled economies may be related to differences in a number of economic components. The GF-GTI slope coefficients modify by nation, displaying that the GF-GTI tie is impacted by the degree and sign of economic shocks, containing the distinct place of the nation (boom and recession), which affects GTI during economic movement [31]. During economic movement, the degree and sign of these shocks impact GTI. In a boom, when the economy is expanding and experiencing growth, the effect of GF on GTI could be diverse compared to a recession, when the economy is contracting and facing economic challenges. During a boom, there may be more resources and investment available for GTI, while in a recession, there may be constraints on funding and a focus on economic recovery rather than innovation. By examining each nation individually, we can explore the specific contextual factors that may impact the connection between GF and GTI. These factors may include the country's economic structure, technological capabilities, regulatory frameworks, investment policies, and cultural attitudes toward sustainable development. Analyzing these nation-specific conditions allows for a more detailed understanding of how GF initiatives interact with the promotion and adoption of GTI. By adopting a country-specific approach, we can identify country-specific patterns and trends regarding the relationship between GF and GTI. Some countries may demonstrate a stronger correlation, suggesting that increased investment in GF positively influences the development and adoption of GTI. In contrast, other countries may exhibit weaker associations due to varying levels of financial support, technological capacity, or policy frameworks. Therefore, we conducted a thorough and comprehensive analysis of each individual nation to gain a complete understanding of its distinct peculiarities.

## 7 Conclusion and policy implications

This work has investigated the non-linear GF-GTI nexus in the selected EU countries (Germany, Sweden, France, Italy, Netherlands, Spain, Denmark, Norway, Belgium, and Finland). The research has applied QQ method to probe dependency between variables by presenting international yet economy-specific intuitions on the GTI-GF nexus. Estimates demonstrated that GF has a positive association with GTI at numerous data distribution quantiles in the majority of our chosen EU economies. However, Spain and Italy show a mixed association.

In light of findings on the GF-GTI nexus within selected EU countries, it is imperative for policymakers to reinforce GF incentives, especially in Germany, Sweden, France, Netherlands, Denmark, Norway, Belgium, and Finland, where a strong positive association was observed. This could involve tax benefits, subsidies, or reduced interest rates for green technology investments. For Spain and Italy, where the association was mixed, a tailored approach is necessary. Their policies should aim at identifying and bolstering specific sectors where GF can most effectively stimulate GTI. These strategies will not only foster sustainable technological advancements but also align with broader environmental goals and economic growth in these regions. The governments should conduct long-term preparation to dedicate greater funds to green products over a long-time horizon. Even if GF does not immediately result in green innovation, it can help GTI progress in the future. Moreover, governments in low-GF nations must be taught from the experiences of high-GF economies and implement more supportive policy frameworks, like tax concessions, to encourage financial capital to stream into clean products. Furthermore, countries with low levels of GF should establish the long-term sustainability of their GF policies to safeguard financial support for GTI because GF encourages more GTI in these nations, and green shareholders may face certain regulatory difficulties.

According to Kudratova et al. [6], profits from green initiatives that assist sustainable development are higher than profits from conventional enterprises. As a result, organizations that are concerned about environmental issues should invest more in R&D for GTI, as well as commit more monetary resources to green procedures that assist in enhancing environmental quality. As environmental preservation is a global priority, investors should seize this opportunity to prioritize GF and GTI by engaging more in GB, green trusts, green leases, and green funds that really can provide higher returns in the future. Precisely, while investors choose to support a green technology program, they should examine it from a long-term view, as this is a proper way to get benefits from GTI. Asset managers should commit more assets and resources to GF, like green funds and GB, which may be used to support GTI and environmental conservation. Asset managers should recognize the weight and share of GF, and the profit rates of such GF should be evaluated over the long-run period.

Because green development is still in its early stages, authorities should exercise strong control over manufacturing activities with more negative environmental consequences and encourage corporations to increase their green innovations in order to operate in an environmentally friendly way. As GF in Italy and Spain has mixed association and the regions which show a negative impact of GF on GTI may be due to the misuse or misallocation of GF funding. Regulators should put more effort into enhancing GF allocation and efficiency by utilizing digital technology to monitor resource consumption to spur future environment protection innovation. Furthermore, corporations should spend more long-term human and financial capital on green technology R&D operations, such as establishing a GTI research department or establishing specific financing for green technologies, which may improve their long-term competitiveness and sustainability. Similarly, corporations can introduce green corporate bonds and expose long-term green investors to the financial market to acquire additional particular financing to boost green technology.

Our study has a few shortcomings that will guide upcoming research on this topic. This research stresses a specific group of economies (EU economies). Altering a group of economies or zones might have a significant influence on the estimates. Consequently, more research in contexts of Sub-Sahara Africa, Asia, the Middle East, the Pacific Region, and emerging nations would aid in improved understanding of the GF-GTI association. A research method that unites household interviews, survey data, big data (if possible), and fieldwork to explore progress on the subject would offer critical information for scientific and administrative groups in identifying the influence of GF on GTI, together with assitance to to government policy actions to ensure a sustainable environment and green technological advancement. Furthermore, more work is required to appraise the influence of GF over other economic variables (including energy efficiency, financial stability, economic growth, and environmental quality).

## Human and animal rights

No animals or human were harmed to do our investigation.

## Availability of data

The datasets utilized throughout the present research are available from the corresponding author on reasonable request.

## Funding

No funding was received for performing current investigation.

## Ethics approval and consent to participate

N/A.

## Consent for publication

N/A.

## CRediT authorship contribution statement

**Jian Yuan:** Writing – original draft, Formal analysis, Data curation. **Sajid Ali:** Writing – original draft, Data curation, Conceptualization. **Raima Nazar:** Writing – original draft, Writing – review & editing. **Muhammad Imdad Ullah:** Writing – review & editing, Writing – original draft.

## Declaration of competing interest

The authors declare that they have no known competing financial interests or personal relationships that could have appeared to influence the work reported in this paper.

## Footnotes

1 Website of Organization for Economic Co-operation and Development (OECD) statistics: https://stats.oecd.org/.
